# A pulmonary artery pseudoaneurysm caused by concurrent chemoradiation therapy for lung cancer

**DOI:** 10.12669/pjms.311.6001

**Published:** 2015

**Authors:** Jung Ho Kim, Sang Hoon Han

**Affiliations:** 1 Jung Ho Kim, Department of Internal Medicine, Gachon University Gil Medical Center, Incheon, Korea.; 2 Sang Hoon Han, Department of Internal Medicine, Jeju National University Institute of Medical Science, Jeju National University School of Medicine, Jeju, Korea.

**Keywords:** Aneurysm, Concurrent chemoradiation, Lung cancer, Pulmonary artery

## Abstract

Pulmonary artery pseudoaneurysms are rarely associated with lung cancer, and to our knowledge, have never been reported as a complication of concurrent chemoradiation therapy for lung cancer. We here report on a 64-year-old man with stage IIIA lung cancer who achieved partial response with concurrent chemoradiation therapy. However, 11 weeks later, he presented with massive hemoptysis, and computed tomography revealed a pulmonary artery pseudoaneurysm. From the findings of this case, we conclude that a pulmonary artery pseudoaneurysm, although rare, warrants attention when concurrent chemoradiation therapy is performed for lung tumors adjacent to the pulmonary artery.

## INTRODUCTION

An aneurysm is clinically defined as a blood-filled sac resulting from arterial or venous wall dilatation. A true aneurysm is a segmental dilatation of blood vessels, including all wall layers, whereas a pseudoaneurysm represents a collection of blood outside the vessel wall, contained by the surrounding tissues.^1^^,^^2^ Pulmonary artery pseudoaneurysm (PAP) and aneurysm are rare, but fatal, disorders. Predisposing conditions include trauma, infection, heart diseases, connective tissue disorders, and vasculitis.^1^

Reports of PAPs related to an adjacent cancer are rare. Specifically, only a few PAP cases have been described in lung cancer patients,^3^^-^^6^ and, to our knowledge, PAP has never been reported as a complication of concurrent chemoradiation therapy (CCRT). Herein, we present the first case of a PAP after CCRT for lung cancer.

## CASE REPORT

**Fig.1:**
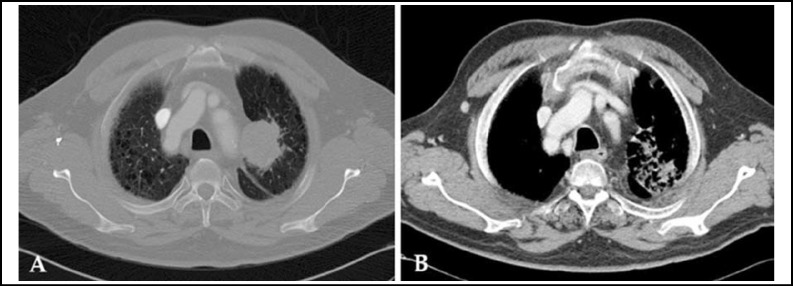
Chest computed tomography findings

**Fig.2:**
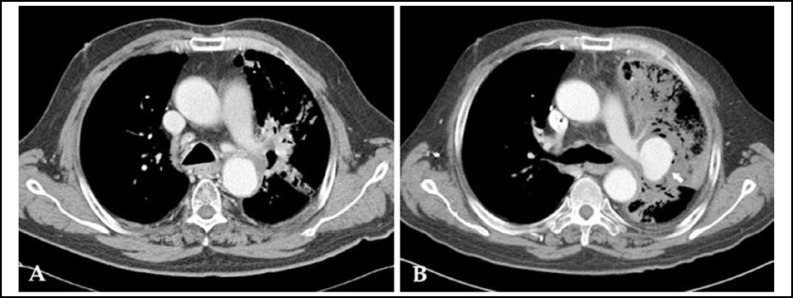
Computed tomography findings of the pulmonary artery before and after the occurrence of the pulmonary pseudoaneurysm

A 64-year-old man with a history of chronic obstructive pulmonary disease and hypertension was diagnosed with non-small cell lung cancer in May 2011. Chest computed tomography (CT) at diagnosis revealed a 4.6-cm mass, later identified as squamous cell carcinoma via biopsy, in the left upper lobe of the lungs (Fig.1A). As the cT4N1M0, stage IIIA tumor was unresectable, CCRT was indicated for a total dose of 68 Gy (2 Gy/day). Concurrently, paclitaxel (50 mg/m^2^) and carboplatin (area under the curve: 2) were administered weekly for 7 cycles. The patient achieved a partial response (Fig.1B). Additional chemotherapy was considered but was delayed at the patient’s request, because of general weakness. In September 2011, he visited the emergency room because of dyspnea and hemoptysis (approximately 100 mL), and decreased hemoglobin levels were noted. CT revealed a 4.8 × 3.2 cm PAP connected to the left upper lobar pulmonary artery (Fig.2).

Lobectomy or pneumonectomy to prevent PAP rupture was not deemed appropriate because of the expected dense tissue adhesion and possible anatomic changes after radiation, and our patient was not eligible for total embolization of the pulmonary artery because of his poor performance status and decreased respiratory capacity. Therefore, only conservative care with antibiotics was provided for aspiration pneumonia due to uncontrolled hemoptysis. The patient died at the end of the pneumonia treatment.

## DISCUSSION

Few reports have been published on cancer-related PAP, and all of them described cases of spontaneous PAPs arising in aggressive lung squamous cell carcinoma as a result of necrosis and vascular destruction.^3^^,^^5^^,^^7^^,^^8^ In our case, however, the PAP developed in a cancer mass with a partial response to CCRT, and there was no evidence of PAP at the initial lung cancer diagnosis or before CCRT. The patient had not undergone previous thoracic surgery, had no history of trauma, and did not have recent tuberculosis infection. Therefore, vascular injury due to CCRT was suspected to be the cause of the PAP.

Treatment-related PAP due to non-surgical injury such as radiofrequency ablation (RFA) has been previously reported. Although considered safe and minimally invasive for the local control of primary or metastatic lung neoplasms, RFA is frequently associated with fatal complications owing to challenges of inserting the RFA probe into major vessels.^6^^,^^9^^,^^10^ Moreover, Chawla *et al.*^11^ reported a PAP case after endobronchial brachytherapy, which is potentially dangerous because of the high doses of local radiation used, resulting in complications such as massive hemoptysis or mediastinal fistula. 

CT angiography, which can precisely identify the extent of a PAP and its related mass, is employed by most institutions for PAP diagnosis.^12^ PAPs typically appear as enhanced round lung masses, isodense to the central pulmonary artery. On contrast-enhanced CT, they appear the well enhanced lesion within the mass. Although the mechanisms underlying vascular injury after radiation therapy are not fully established, various factors, including early atherosclerosis, vasa vasorum degeneration, and arterial wall necrosis, have been suggested.^13^ Fonkalsrud *et al.*^14^ suggested that injury to vascular endothelial cells begins 48 hours post-radiation, and is followed by bleeding, tunica media necrosis, and fibrosis, consequently leading to vessel structure morphologic changes. Subsequently, ischemic changes caused by the injured vasa vasorum and tunica adventitia fibrosis may induce large vessel rupture and PAP formation.

PAP-associated symptoms vary according to its size and onset conditions. Patients with PAP due to sudden trauma often experience hypovolemic shock. Conversely, chronic PAP is usually asymptomatic during its early phase, and chest pain, cough, hemoptysis, recurrent pneumonia, and dyspnea are commonly observed as it progresses. In our case, since the interval between the CT on which PAP was detected and the previous CT was 11 weeks, the PAP probably formed owing to vascular connective tissue and endothelium injuries by CCRT, progressed gradually, and ultimately led to massive hemoptysis.

Small PAP lesions under low pressure often resolve spontaneously.^15^ However, urgent intervention is required for life-threatening massive hemoptysis. In our case, surgical management with, for example, pneumonectomy was deemed unsuitable owing to the expected dense adhesion and pulmonary vasculature friability after CCRT, and the high risk of vascular and bronchial stump rupture, even after successful surgery, outweighs any potential benefits. Moreover, pulmonary artery embolization could not be performed because of the patient’s poor pulmonary function and high risk of hypoxia, and coil embolization, a symptom management procedure to prevent PAP rupture, was deemed unsuitable considering the pulmonary infarction risk.

To our knowledge, this is the first report of a PAP as a complication of lung cancer CCRT. Although its prevalence remains unclear, PAP should be recognized as a potentially serious complication in patients receiving CCRT for lung tumors adjacent to the pulmonary artery.

**(A)** At the initial cancer diagnosis, a 4.6-cm mass adjacent to the pulmonary artery was observed in the left upper lobe of the lungs.

**(B)** The patient achieved a partial response to concurrent chemoradiation therapy, as evidenced by tumor shrinkage.

**(A) **After concurrent chemoradiation therapy (CCRT), the tumor markedly decreased in size, and some necrotic tissue was observed. However, there was no evidence of a pseudoaneurysm.

## Notes

***Conflict of*** ***interest*** ***:*** The authors state that they have no Conflict of Interest (COI).
